# Scientific output scales with resources. A comparison of US and European universities

**DOI:** 10.1371/journal.pone.0223415

**Published:** 2019-10-15

**Authors:** Benedetto Lepori, Aldo Geuna, Antonietta Mira

**Affiliations:** 1 Faculty of Communication Sciences, Università della Svizzera Italiana, Lugano, Switzerland; 2 Department of Economics and Statistics Cognetti De Martiis, University of Turin, Turin, Italy; 3 BRICK, Collegio Carlo Alberto, Turin, Italy; 4 Institute of Computational Sciences, Faculty of Economics, Università della Svizzera Italiana, Lugano, Switzerland; KU Leuven, BELGIUM

## Abstract

By using a comprehensive dataset of US and European universities, we demonstrate super-linear scaling between university revenues and their volume of publications and (field-normalized) citations. We show that this relationship holds both in the US and in Europe. In terms of resources, our data show that three characteristics differentiate the US system: (1) a significantly higher level of resources for the entire system, (2) a clearer distinction between education-oriented institutions and doctoral universities and (3) a higher concentration of resources among doctoral universities. Accordingly, a group of US universities receive a much larger amount of resources and have a far higher number of publications and citations when compared to their European counterparts. These results demonstrate empirically that international rankings are by and large richness measures and, therefore, can be interpreted only by introducing a measure of resources. Implications for public policies and institutional evaluation are finally discussed.

## Introduction

During the last thirty or so years, public funding of research institutions and particularly of universities [1] has significantly changed, moving from a largely historical allocation based on the presumption that society will reap the benefit of science [2] to an evaluative culture where resources are increasingly distributed based on some measure of performance [3]. These changes signal a move from a conception of science as a ‘public good’ [4] to a conception of science as a commodity whose supply is governed by market mechanisms [5].

Such new conception of science is grounded on the belief that there are ‘universal’ measures of scientific ‘excellence’ that academic managers, policymakers and stakeholders can reliably use to assess the output of individual researchers and universities. A continuously evolving family of bibliometric indicators have been generated and used/misused both at the micro management level, to take decisions on salaries and career, and at the macro level, directly or indirectly, in performance-based university funding systems [6]. In parallel, during the last fifteen years, academic rankings—closely associated to the same bibliometric measures—moved to the center stage of public debate on science [7]. Though often criticized for their intrinsic limitations, rankings have been used to provide so-called ‘excellence’ signals to stakeholders, e.g. private donors, companies and international students [8] [9].

Across countries, one model of research intensive institution, inspired by the US research university [10], has become the aspirational archetype for all universities that are increasingly involved in the battle for international ‘excellence’, with university managers keenly scrutinizing their position in international rankings [11]. At the political level, the observation of a ‘transatlantic gap’ in bibliometric indicators between US and Europe [12] has led to a wide debate on whether stronger policies rewarding ‘excellence’ would be needed.

However, such an approach under evaluates the institutional and historical diversity of local higher education institutions (HEIs) with their heterogeneity in missions and responses to specific local needs [13] and generates self-reinforcing cumulative mechanisms epitomized by the Matthew effect where the rich is becoming richer [14]. While such cumulative effects are at the core of the scientific enterprise [4], the acritical use of indicators that are ‘blind’ against diversity risks to create adverse effect such as loss of innovation and of responsiveness to societal needs [15].

At a more technical level, the bibliometric literature has demonstrated the potential flaws of such indicators and, specifically, of using them without benchmarking against the level of available resources [16]. Preferential attachment in visibility, as witnessed by super-linear scaling between the volume of publications and of citations in the case of countries, scientific fields [17], cities [18] and universities [19], implies that so-called ‘scale-free’ indicators such as Mean-Normalized Citation Scores (MNCS [20]) in reality are size dependent. In a systematic criticism, Abramo and D’Angelo argue that bibliometric indicators cannot be used as reliable signals for evaluation and allocation of resources because they do not take into account the amount of resources invested [21].

Therefore, the new governance of science suffers of two main shortcomings. First, while quantitative research evaluation methods are robust in identifying low performance (in the selected output measurement), they are less reliable when used to discriminate top performance. Moreover, research evaluation has to be carried out in comparative way, comparing apples with apples, and thus requires that inputs are taken into account and alternative outputs are compared. Specifically, and at the core of the contribution of this paper, funding needs to be considered if we want to properly understand productivity of individuals and organizations. Yet, beyond the obvious assumption that more resources translate into more output, we know little about the relationship at the institutional level between the amount of available resources on the one hand and scientific output and visibility on the other hand [22] [23]. All evaluation efforts have been directed toward measuring output rather than productivity [21].

The focus of this paper is to examine the relationship between resources and standard bibliometric indicators that are widely used to compare universities for their ‘excellence’ (for example, in international rankings). We aim to understand whether such indicators depict wealth rather than anything else.

Our contribution is mainly empirical. We first test the association between university revenues and international visibility. Using a dataset providing input and output data for nearly all doctoral universities in the US and in Europe, we demonstrate that the number of publications and citations at the university level scales super-linearly in respect to revenues, and that these relationships are similar in the US and in Europe. This implies that the richest universities will systematically show up at the top of bibliometric indicators and of international rankings. Second, we show that the main path associating university revenues with bibliometric output is through additional revenues per staff, suggesting that the wealthiest universities compete for talented researchers by offering them more attractive funding packages [2].

Third, we investigate to which extent such differences between US and Europe in international visibility are associated with different levels and distribution of resources by HEI. We show that the US system comprises a small number of universities with far larger revenues than their European counterparts. This suggests that the ‘transatlantic gap’ in research ‘excellence’ [12] is by and large a ‘resources gap’ and the outcome of a resourcing model that concentrates a large amount of resources in a few universities independently from student enrolments.

Finally, from these results we derive implications for evaluation practices, academic management and public policies. Our results support claims from the evaluation community, such as expressed in the DORA-San Francisco Declaration ([24]) and in the Leiden Manifesto [15], that performance needs to be evaluated according to the different objectives attributed to HEIs, but also to the league in which an HEI competes, which is largely defined by the level of resources available. Since our results demonstrate how strong and pervasive cumulative effects are concentrating resources in the internationally excellent universities, public policies should focus in fostering institutional diversity and responsiveness to societal demands rather than in priming the richest universities. In turn, institutional managers should not attempt to imitate the top-ranked institutions, since this would be hardly possible given the huge differences in available resources, but rather to identify a specific ‘quality’ niche and to compete with other institutions with a similar level of resources.

## Materials and methods

**Fig 1:**
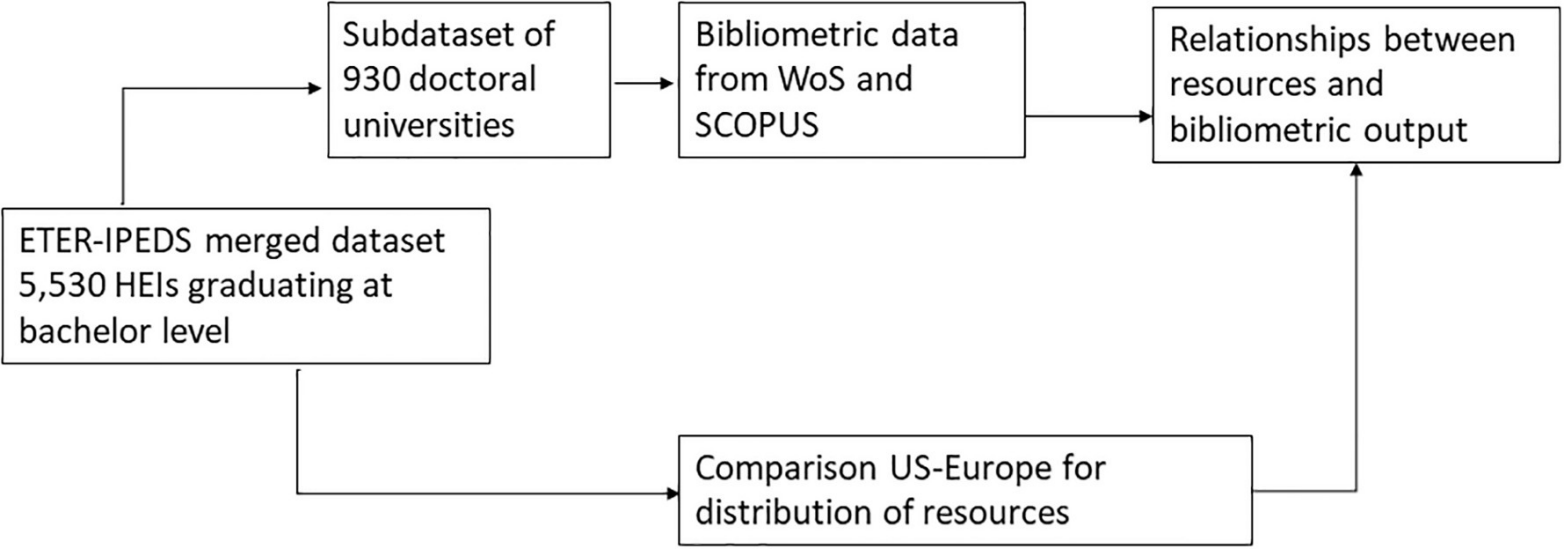
Empirical strategy.

Our empirical strategy is described in Fig 1.

First, we have created a dataset including the full population of HEIs delivering at least a bachelor degree in the two systems (excluding associate colleges in the US), i.e. 3,287 HEIs in the US and 2,243 HEIs in Europe. Data have been derived from the Integrated Postsecondary Education Data System for the US (IPEDS; [25]) and the European Tertiary Education Register database (ETER; [26]). When compared with international student statistics from EUROSTAT, the coverage of our dataset is 100% of student enrolments at bachelor, master and PhD level in the US and 96% in Europe.

Second, from this dataset, we have extracted the subpopulation of *doctoral universities*, defined as the HEIs awarding more than 20 PhD degrees in the reference year 2013 and excluding universities focused on a single topic such as medical schools (the criteria adopted by the US Carnegie classification; [27]). This subpopulation is composed of 564 universities in Europe and 366 universities in the US. It includes 22 out the top-25 and 77 out of the top-100 universities in the ARWU ranking (2017 edition), the remaining being in other regions worldwide, and is therefore highly representative of what is considered as international research ‘excellence’. We use this subpopulation, first, to analyze the relationship between the volume of research and bibliometric outputs (publications and field normalized citations) and, second, to analyze the path linking revenues, staff and outputs through a mediation model. Bibliometric data were extracted from the Web of Science copy at CWTS, Leiden University, and from Scopus-SCIMAGO in a robustness check.

Third, we use the full dataset to compare the volume and distribution of revenues within the two systems and to examine to which extent this accounts for differences in resourcing of doctoral universities between US and Europe.

### Variables

Table 1 provides an overview of the variables used in the paper. The reference year is 2013 for all variables except for publications and citations, which refer to the period 2014–2017 to allow for a time-lag between input and output.

**Table 1:** Variables, valid and missing cases.

Variable Name	Definition	Valid cases US	Valid cases Europe	Missing cases	Missing (doctoral universities)
*Classification variables (Carnegie classification)*					
Highest Degree Delivered	1 = bachelor (3 or 4 years); 2 = master or equivalent diploma in the pre-Bologna system (for example 4/5 years license); 8 = doctorate.	3,287	2,170	81	0
Degrees by level	Number of degrees awarded by level of degree.	3,054	2,049	435	0
Subject composition	Herfindahl index of the distribution of students by field using the fields of education and training classification.	3,056	1,980	502	0
*Relationships between revenues and output*					
Total current revenues	Total Revenues euro PPP (excluding hospital revenues and subsidiaries)	3,062	1,270	1,206	142
Academic staff	ETER = academic staff. IPEDS = instructional, research and public service staff, both in Full Time Equivalents	3,195	1,708	627	114
Students	Total number of students enrolled at bachelor, master and PhD.	3,150	2,243	137	0
Students in SSH	Share of students in education, humanities and arts, social sciences, business and law.	3,056	1,989	487	32
Publications	Publications count (Web of Science).	440	850	4,240	27
Normalized citations	Field normalized citations count.	440	850	4,240	27
Region	Dummy variable: US vs. Europe.	3,287	2,243	0	0
*Analysis of HEIs revenue structure*					
Basic state installment	State allocation for the general functioning of the university.	2,570	437	2,523	395
Private donations and endowment	Private donations and revenues from the endowment attributed to the university as a whole.	2,570	436	2,524	372
Third Party Funding	Public and private contracts, including those from public agencies (NSF etc.).	3,062	1,163	1,305	171
Student fees funding	Fees paid by students, including also indirect state support (for example loans).	3,062	1,136	1,332	175
Legal Status	0 public institutions (IPEDS = public, ETER = public or private government-dependent); 1 private institutions (IPEDS = private for profit or private non-profit, ETER = private)	3,287	2,240	3	0

The first group of variables is used to classify HEIs based on the Carnegie classification criteria and to identify doctoral universities in our population (see S1 Text. Applying the Carnegie classification to our samplefor the application of the Carnegie classification to our sample).

The second group of variables is used for the analysis of the relationship between revenues and bibliometric outputs.

Finally, the division of revenues by streams is used for a comparative analysis of HEI resource structure; legal status is in this respect an important control factor as the resource structure differs between public and private HEIs.

For the classificatory variables, the number of missing cases is below 10%; accordingly, only 6% of the HEIs, corresponding to 1% of academic staff and students, could not be classified. For the regression variables, missing cases reduce the regression sample from 930 to 751 cases, mostly because of missing data in total current revenues for European universities. However, the valid cases still produce 91% of the publication output and include 76 (instead of 77) out of the top-100 universities in the 2017 ARWU-Shanghai ranking.

Coverage of revenues data is lower due to missing data in Europe; however, data availability is much higher for doctoral universities, which are the main focus of the analysis. For most European universities, the resources for the general functioning of the university cannot be broken down between basic state instalment and private donations; since available data show that the share of private funds is low in Europe, we use simple imputation based on the average of available cases (in Europe) to complete the dataset.

*Total Current Revenues* are the amount of money received by the HEI during the reference period for its operations. It excludes revenues intended for long-term investment, such as state subsidies for buildings and large facilities. Excluding capital-related revenues is important for comparability purposes due to different treatments of capital costs and revenues depending on the university accounting system (usually cash accounting for public HEIs and accrual accounting for private HEIs). Investment income (for example revenues generated from assets and endowments) is included. Revenues from ancillary enterprises are also excluded. This is important since, for US universities, sales and services from auxiliary enterprises and intercollegiate athletics might constitute a large share of total revenues. Finally, revenues of university hospitals are excluded, but educational and research costs of the medical faculties are included.

For a more fine-grained analysis, *total current revenues* are divided into four streams:

- *Basic state instalment*, i.e. the funds provided by the state for the general functioning of the HEI.
- *Private donations and pay-outs from the endowments*, managed at the university level.
- *Third-party funds* mostly for research, e.g. research grants from public funding agencies and contracts from companies.
- *Funding from student fees* paid by students and families.

To this aim, we devised a mapping scheme based on the revenues subcategories provided by IPEDS and ETER (see S1 Table. Mapping scheme of HEI revenues). Such a disaggregated approach allows a more precise control of the revenue perimeter and of comparability problems.

For all financial variables, we use Purchasing Power Parities in euros from Eurostat, as they take into account cost differences between countries. Since PPPs for the US are below one (1 US $ = 0.734 euros), this somewhat reduces funding level differences between the US and Europe.

*Academic Staff* in Full Time Equivalents are based on working contracts; in ETER, all personnel involved in teaching and research is included, while for IPEDS, we use the number of instructional, research and public service staff as the nearest equivalent. In both cases, it excludes management, technical and support staff, as well as healthcare staff in the hospitals annexed to universities. Coverage of PhD students and postgraduate staff may not be fully complete. However, when using FTEs, this is less of a concern if part-time staff is not fully covered.

*Publications* and *Normalized Citations* are derived from the Web of Science (WoS) copy maintained by the CWTS, University of Leiden, which is also the source of the Leiden ranking [28]. The list of HEIs in our dataset has been matched with the institution list in this dataset: this process was straightforward thanks to the extensive standardization of institution names in the Leiden WoS copy, dubious cases could be resolved by using information on the website and location. Given the small number of institutions involved, the matching was performed manually.

The Leiden ranking includes a substantial effort to delineate the perimeter of universities and to handle special cases, e.g. assigning publications correctly to members of confederate universities (e.g. University of London). University publications also include university hospitals, which are tightly integrated with the university, as revealed by publications with shared affiliations. This avoids comparability issues between situations where the hospital is part of the university (as in many US universities) or it is legally independent (as in most European countries).

Bibliometric data were retrieved for 903 out of 930 doctoral universities. Most missing cases were special institutions such as distance graduate schools in the US.

The indicators follow the definitions adopted for the Leiden Ranking [29]:

- The count of publications (P) includes only the core publications in the WoS, i.e. those published in journals of international scope and highly referenced in the WoS. This is consistent with our focus on international research ‘excellence’. Fractional counting of publications was adopted. The reference period is 2014–2017.
- The total normalized citation score (TNCS) is the total number of citations of the publications normalized for field and publication year. The citation window is of variable length depending on the year of publication since citations are included only up to week 13.2019. Citations are also fractionalized.

We carried out a robustness check of our main results using the other main international bibliometric database, i.e. Scopus. The list of doctoral universities was matched with the SCIMAGO Institutional Ranking (SIR). We were able to identify 867 out of 930 doctoral universities in the SIR. Most non-matched cases were either small institutions or multi-campus HEIs in the US, for which SIR only provided aggregated data.

A well-known issue for bibliometric data are differences in publication behavior and database coverage across scientific fields; since universities have different subject compositions, this might weaken the observed relationship, as extensively analyzed in [19]. We have partially controlled for this effect by excluding from our sample mono-disciplinary HEIs, such as medical schools and by using field-normalized citation scores to partially account for differences in citation patterns. Further, we have added in the regressions a control for the share of students in social sciences and humanities. Finally, the robustness check with Scopus data is indicative that our results are not sensitive to database coverage, since Scopus includes more social sciences journals and books that the WoS.

### Methods

First, to test the association between revenues and bibliometric outputs, we regress the two bibliometric indicators, i.e. the number of publications and of field-normalized citations, against total university revenues (in euros PPPs). Since data refer to total revenues, including educational expenditures, we also control for the volume of education and for the share of students in social sciences and humanities, while we introduce a dummy for US vs. Europe in order to control for data comparability issues.

The standard approach for fitting power-law relationships is to use an OLS regression on the log-transformed variables and to provide an analysis of residuals to check whether there are potential robustness issues [30]. Two major concerns are heteroscedasticity, since the variance is larger for smaller HEIs, and non-linearity, i.e. scaling coefficients not being constant over the whole range of the dependent variable.

As a first step, we use OLS to detect and to exclude influential observations and outliers. Then we address heteroscedasticity through Feasible Generalized Least Squares (FGLS [31]). FGLS is an estimator correcting for heteroscedasticity by estimating weights for the variance from an analysis of OLS residuals and then using them in the regression to estimate coefficients and standard errors.

First, the following OLS regression is estimated:

ln(publications)=αln(revenues)+βln(students)+γ(socialsciencesstudents)+δ(region)+ε (1)

Then, regressing the squared residuals of Eq (1)

$$\text{ln}\left(\text{residual}2\right)=\alpha_{1}\;\text{ln}\left(\text{revenues}\right)+\beta_{1}\;\text{ln}\left(\text{students}\right)+\gamma_{1}\;\left(\text{social}\;\text{sciences}\;\text{students}\right)+\delta_{1}\;\left(\text{region}\right)+\varepsilon$$ (2)

Weights are computed from the predictions of Eq (2) and then used in a weighted OLS regression:

$$w_{i}=\text{exp}\left(predicted_{i}\right)$$ (3)

$$\text{ln}\left(\text{publications}\right)=\alpha_{2}\;\text{ln}\left(\text{revenues}\right)+\beta_{2}\;\text{ln}\left(\text{students}\right)+\gamma_{2}\left(\text{social}\;\text{sciences}\;\text{students}\right)+\delta_{2}\left(\text{region}\right)+\varepsilon$$ (4)

where

$${\text{var}}_{\text{i}}=\sigma^{2}{\text{w}}_{\text{i}}$$

so that observations with large predicted residuals are given less weight in the estimation. Model diagnostics shows that, for our data, this model strongly improves the structure of residuals (see S2 Text. *Model diagnostics and robustness tests*). Complementarily, we perform quantile regressions [32], which allow investigating linearity, i.e. the extent the observed effect differs by the level of the dependent variable [30].

**Fig 2:**
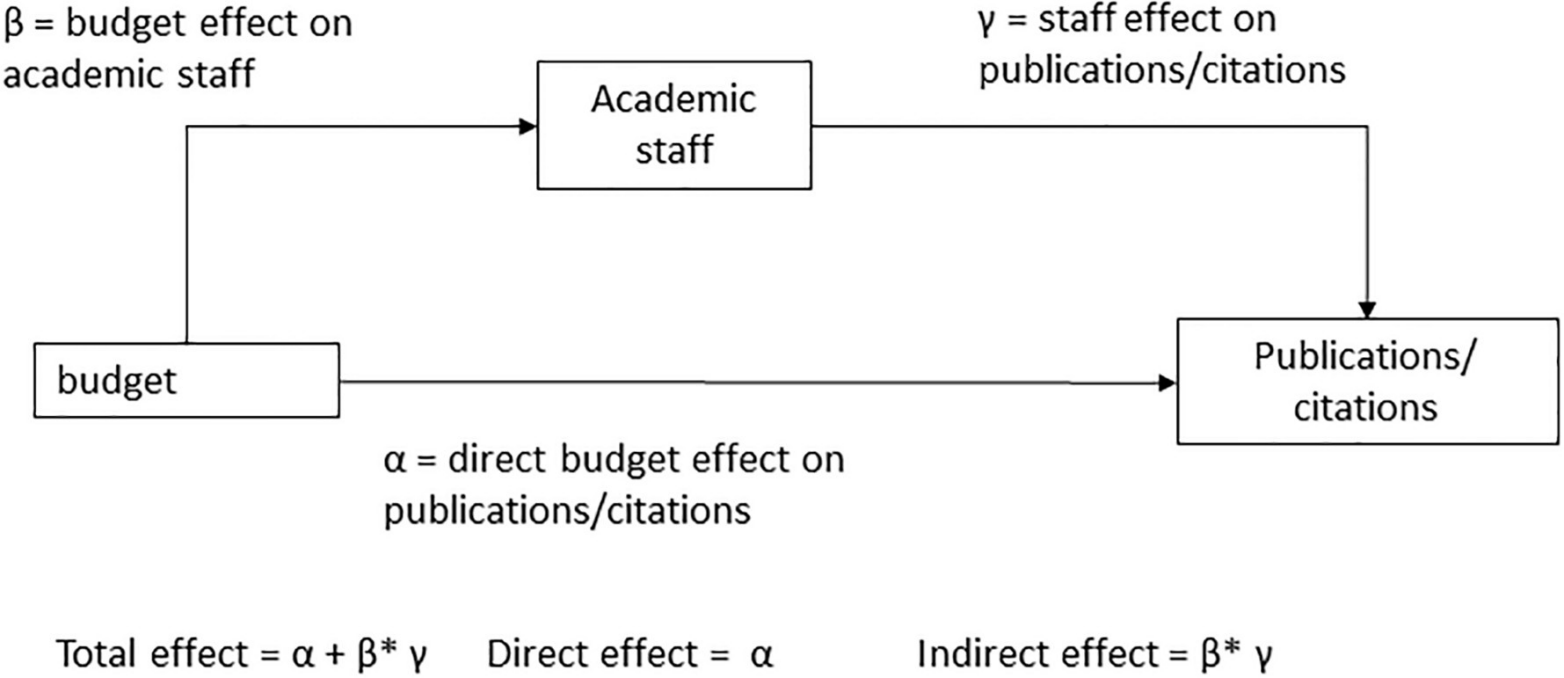
Mediation model.

Second, to test association paths between revenues, staff and bibliometric output, we run a mediation model that allows estimating the paths that associate revenues and publications (respectively field-normalized citations) directly or through the number of academic staff (Fig 2). Mediation models are relevant when two variables, e.g. revenues and staff are strongly correlated and both are expected to affect an outcome variable, i.e. bibliometric indicators [33].

Third, to compare the level and the composition of revenues between US and Europe, we perform descriptive analyses using the full sample of 5,530 HEIs. We compare the aggregated level of revenues in the two systems, as well as the distribution between HEIs. Then, we analyse the distribution of total current revenues between subcategories. Finally, we compare the distribution of revenues, staff and bibliometric outputs among the sample of 930 doctoral universities to ascertain whether we observe differences in concentration of revenues and outputs between US and Europe.

## Results

### Scaling properties of bibliometric output

As reported in Table 2, a linear relationship is observed on the log-log scale between universities revenues and bibliometric output, with slope 1.46 for publications (p-value < 0.001) and 1.67 for field-normalized citations (p-value < 0.001), corresponding to the degree of the power law distribution for publications and field-normalized citations over revenues.

**Table 2:** Regression results for publications and citations, FGLS regression.

	ln(publications)			ln(citations)		
	Coef.	Std. Err.	p	Coef.	Std. Err.	p
Ln(revenues)	1.457	0.035	0.000	1.686	0.039	0.000
Ln(students)	0.023	0.048	0.627	-0.118	0.052	0.025
Share students SSH	-0.817	0.171	0.000	-0.660	0.186	0.000
region	-1.103	0.060	0.000	-1.259	0.066	0.000
_cons	-19.464	0.509	0.000	-22.370	0.561	0.000
N	751			750		
R-squared	0.805			0.807		

These findings have important implications for the use of bibliometric indicators for evaluation purposes. On the one hand, the coupling between revenues and bibliometric indicators is really tight, as shown by the coefficient of determination. On the other hand, super-linear scaling implies that bibliometric indicators increase more rapidly than revenues and so-called scale-free indicators, such as MNCS, become size-dependent [21]. Such a relationship implies that the position in international rankings is strongly associated with university revenues—16 out of the top-25 US and European universities in the 2017 ARWU-Shanghai ranking are among the top-25 HEIs in our dataset for revenues, and Harvard and Stanford top both lists.

We notice that the regression sample includes a large share of the population of internationally ‘excellent’ universities (76 out of the top-100 in the ARWU-Shanghai ranking) and almost all doctoral universities in the two regions. This emphasizes the significance of our results.

We also run the same regression using data from the other large international bibliometric database, i.e. SCIMAGO, and more specifically the number of documents for the period 2015–2017 and the field-normalized citation impact. Results are very similar to the WoS (see S2 Text. *Model diagnostics and robustness tests for full results*), confirming previous results that, indicators from WoS and Scopus tend to be highly correlated at aggregation levels such as universities or countries [34]. This provides support that our results are not database-specific.

**Fig 3:**
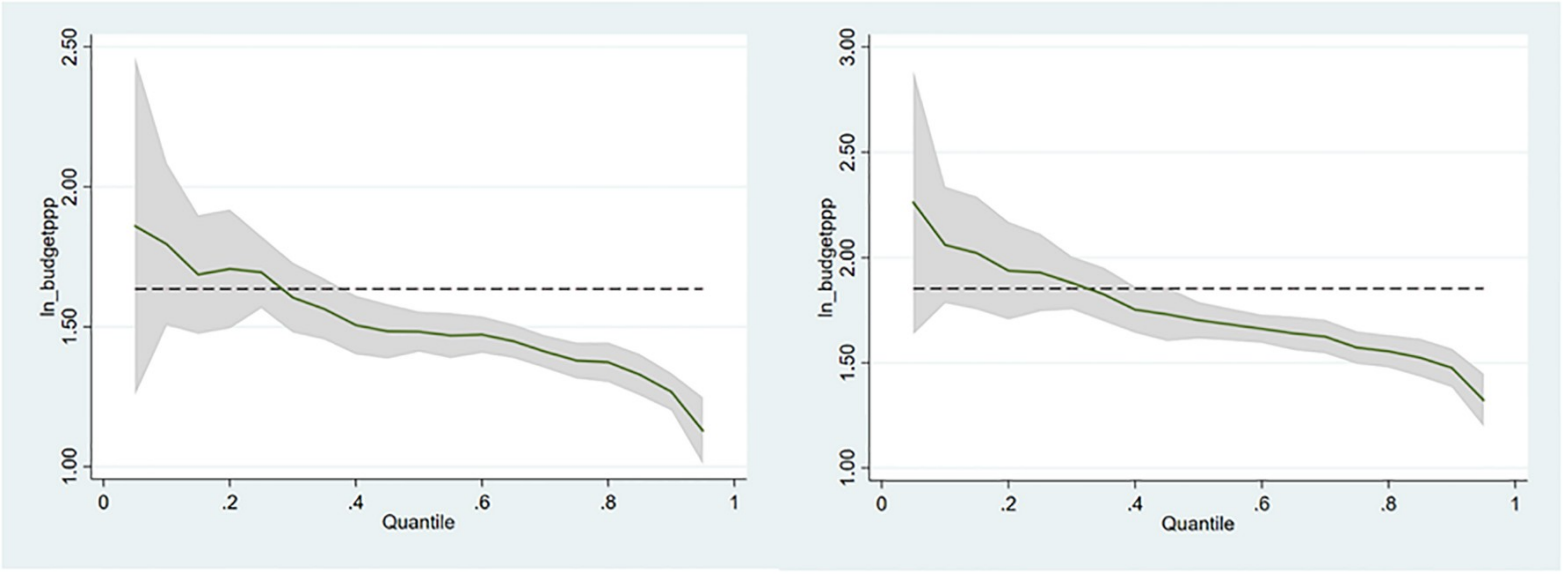
Quantile regressions of ln(revenues) for dependent ln_publications (left) and ln_citations (right). The dashed line corresponds to the OLS estimate, the grey are the coefficient’s SE.

Finally, quantile regressions show that the scaling coefficients decrease with the quantile, but remain significantly above one for the whole range of the dependent variables; the standard error also decreases for higher levels of the dependent, since less variance is expected for large HEIs because of aggregation effects (Fig 3). On the one hand, this implies that scale effects are stronger for the smaller HEIs (as observed also in cities [35]), but remain significant for the top-ranked universities. On the other hand, the coupling between revenues and research outputs is tighter at the top of the pile.

We also perform a number of further robustness tests including separate regressions for the two regions, which produce similar results to the main regression, and an analysis of residuals and outliers; the latter shows that most deviant cases are in the left-tail of the small universities, while the fit is better for the top-ranked international universities (see S2 Text. *Model diagnostics and robustness tests* for diagnostic analysis).

### Revenues, staff and output

Data show a very high correlation between revenues and staff for research universities (0.88 on a log-log scale), as expected since the main resource for universities is academic staff and, therefore, additional funds will be largely invested in hiring people. However, universities could also provide more resources per unit of staff, for example in the form of higher salaries or of starting packages for newly hired professors [2].

As reported in Table 3, the main association between revenues and publication and citation output is through the amount of revenues independent from the number of staff. The direct coefficient of revenues to publications is 1.269, while the indirect coefficient through staff is 0.605*0.673 = 0.407. Both coefficients are statistically significant, but the former accounts for about two-thirds of the total.

**Table 3:** Mediation models for citations and publications (OLS with robust standard errors).

	Ln(publications)			ln(citations)		
Ln(staff)	0.605	0.121	0.000	0.590	0.135	0.000
Ln(revenues)	1.269	0.101	0.000	1.492	0.115	0.000
Ln(students)	-0.311	0.077	0.000	-0.423	0.085	0.000
Region = US	-1.144	0.096	0.000	-1.317	0.105	0.000
_cons	-18.275	1.284	0.000	-21.338	1.434	0.000
	Ln(staff)			Ln(staff)		
Ln(revenues)	0.673	0.022	0.000	0.673	0.022	0.000
Ln(students)	0.235	0.028	0.000	0.235	0.028	0.000
US	-0.505	0.025	0.000	-0.505	0.025	0.000
_cons	-8.031	0.254	0.000	-8.031	0.254	0.000
Indirect coefficient	0.407	0.080	0.000	0.397	0.089	0.000
Direct coefficient	1.269	0.101	0.000	1.492	0.115	0.000
Total coefficient	1.676	0.055	0.000	1.889	0.061	0.000
N	718			718		
AIC	2093.633			2202.884		

As expected, student enrolments have a positive association with the number of staff, implying that, with increasing number of students, revenues are used to a larger extent to hire staff, generating some increase in output. However, the aggregate coefficient is negative (0.235*0.605–0.311) = -0.170, i.e. universities with more students have less publications and citations with the same resources.

These results show that the stronger influence of resources on scientific production takes place through providing more resources per unit of staff, as this allows the richer universities to compete for the most talented researchers. However, increasing student enrolments push universities to expand their staff to manage educational activities and, in turn, this lowers scientific output. Therefore, for international ‘excellence’, not only the amount of resources matters, but also the extent to which revenues are decoupled from the number of students.

### US vs. Europe differences in resource distribution

While scaling relationships are similar, the two systems are characterized by large differences in the distribution and composition of HEI revenues.

**Fig 4:**
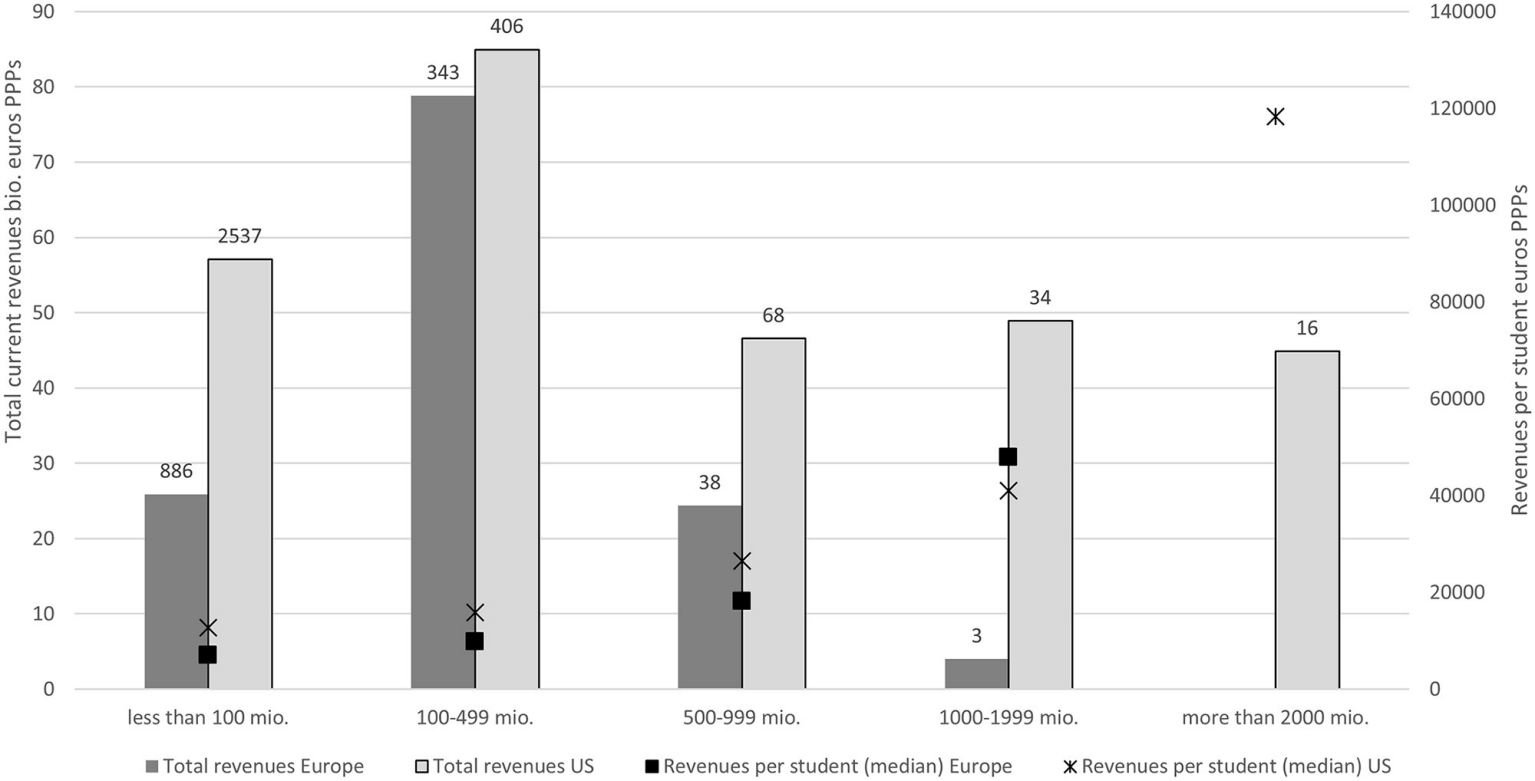
HEI revenue classes. Number of HEIs by region and class. Left axis: sum of revenues by class. Right axis: revenues per student.

Fig 4 shows that the US system includes a larger number of small HEIs and a group of HEIs with extremely large revenues, while in Europe the largest portion of resources are directed to middle-size HEIs. On the top of the pile, the US system includes 16 HEIs with total revenues above 2 billion euros in PPPs, while the 50 HEIs with revenues above 1 billion constitutes one-third of all resources. On the contrary, in Europe there are only 3 HEIs with revenues above 1 billion, while half of the resources are accounted for by middle-sized HEIs below 500 million Euros.

All top-25 HEIs by revenues in the dataset are in the US, with the list being topped by Harvard and Stanford, the first European universities are Cambridge (place 26) and Oxford (place 41), i.e. the highest ranked European HEIs in the 2017 ARWU-Shanghai ranking. Interestingly, HEIs in the same revenue class have similar levels of funding per student in the two systems, showing that the main difference lies in the distribution of revenues and, particularly, in the presence in the US of a group of about two dozens of universities with extremely high revenues.

When combined with super-linear scaling of publications and citations over revenues, the distribution of revenues translates into a dominance of the US universities in the ranking by number of publications and citations (both absolute and normalized by volume), respective in the international rankings, which are closely correlated to bibliometric indicators.

### Institutional differences

A deeper analysis reveals that a combination of institutional factors accounts for the observed difference in the distribution of revenues.

First, the US higher education system is endowed with more resources. When comparing only HEIs that have financial data, the numbers of staff, students, publications and citations are similar in both systems, while the total amount of revenues is 282 billion euros PPS in the US and 133 billion euros in Europe (Table 4), showing how the transatlantic ‘excellence’ gap is essentially a resource gap [12].

**Table 4:** Aggregated data for US and Europe.

		N. of HEIs	Total revenues (million PPPs)	Academic staff FTE	Students ISCED 5–7	Graduates ISCED8	Publications	Citations
All HEIS	Europe	2,243	133,042	962,350	17,087,184	123,604	1,197,146	1,303,339
	US	3,287	282,401	842,730	13,669,196	69,303	937,127	1,177,564
Only HEIs with financial data	Europe	1,270	133,042	798,644	12,928,020	102,547	1,002,987	1,122,954
	US	3,062	282,401	832,116	13,448,559	68,769	931,059	1,170,268

The difference in resources in our data is compatible with international statistics where tertiary education spending was 2.7% of GDP in 2014 in the US and ranged between 1% and 2% in European countries (source: OECD, Education at a Glance). This difference is essentially due to revenues from the private sector and from students (also including state subsidies to students) that account for two-thirds of tertiary education spending in the US, but to less than 40% in most European countries (with the exception of the UK).

Second, we observe a difference in the extent of institutional differentiation between the two systems, as revealed by applying the US Carnegie classification to the dataset (see S1 Text. Applying the Carnegie classification to our sample). Although the European system comprises a large number of colleges and specialized HEIs, doctoral universities account for nearly 70% of academic staff and students at the bachelor and master level, when compared to 55% of staff and 45% of the enrolled students for US doctoral universities. The difference would have been even larger when considering all tertiary education institutions, since HEIs delivering short degrees (associate colleges) are far more important in the US than in Europe.

Since colleges receive fewer resources per student, a higher share of students attending colleges translates into more resources for doctoral universities. This difference has lasting historical roots: the US system was grown from different institutional models, including the appearance of the research university as a distinctive type of institution during the 20^th^ century [36] [37]. Europe was historically dominated by the “Humboldtian” public university model, with attempts to differentiate a second sector of higher education only starting in the 1970s [38].

**Fig 5:**
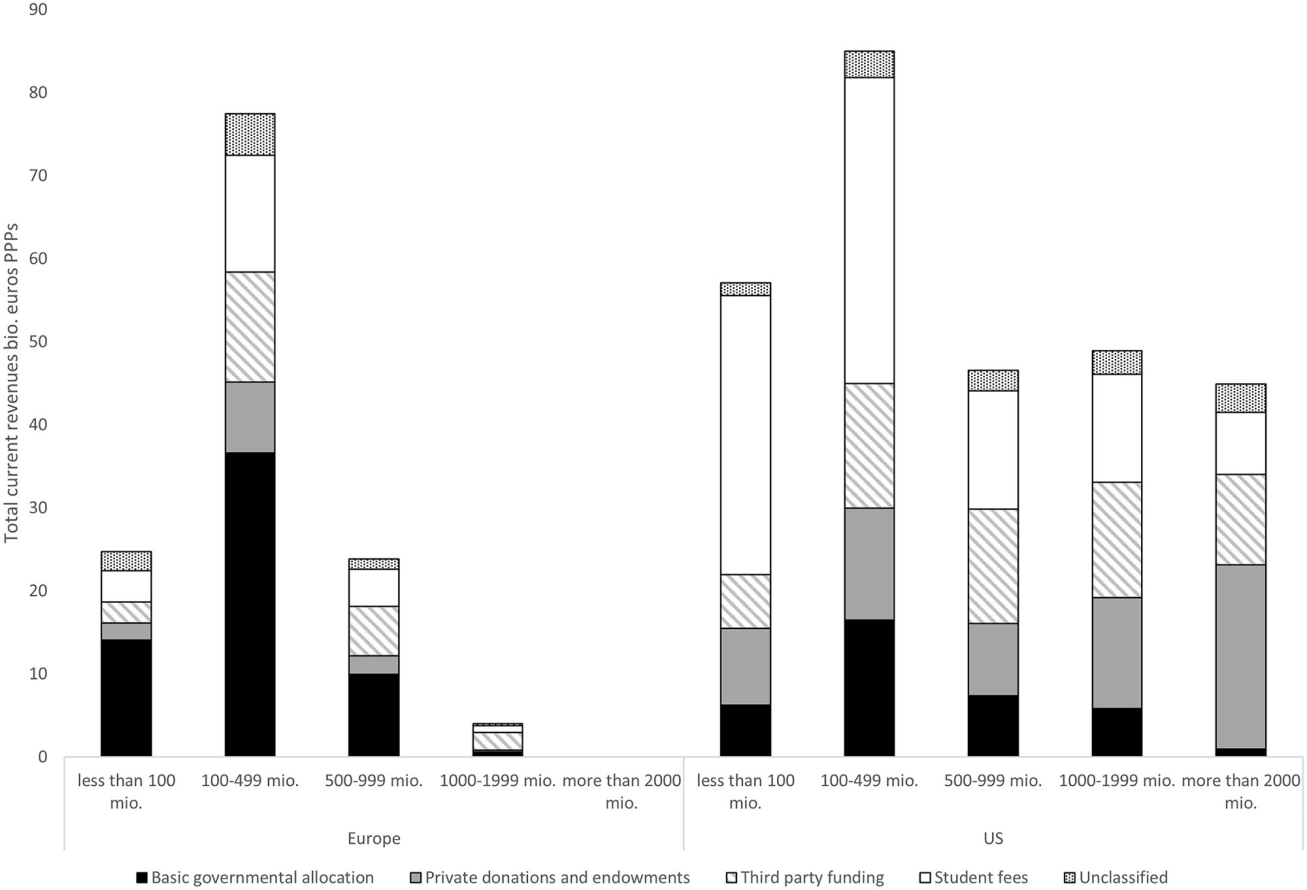
Revenue sources by revenue class. The numbers on the top of the bar are the number of HEIs in each group.

Third, the US system is characterized by a stronger differentiation of revenue sources in the aggregate and between HEIs. As demonstrated in Fig 5, most European HEIs have a funding model where the basic government allocation represents the largest share of funds, while other sources are complementary—the only exceptions are private for-profit HEIs and public UK universities that are mostly funded through student fees.

On the contrary, US universities have a differentiated funding model, where private revenues and student funding play a central role—the latter being largely indirect state support through student loans and subsidies. Differences within the system are large. The public (state) universities have a composite funding structure, in which state funds represent a sizeable (even if diminishing) share of the revenues [39], while private for-profit HEIs are mostly funded by student fees, similar to Europe. Finally, the private non-profit sector, that comprises most of the top-universities in terms of bibliometric output, is funded by a combination of private donations and endowments, and through student fees.

We also observe differences in how the largest universities are funded. In the US, private donations and endowments are the main source for the largest institutions and are heavily concentrated at the top of the pile (Fig 5). The 16 universities with revenues above 2 billion euros receive 53% of the private donations that constitute 49% of their revenues. On the contrary, in Europe, the universities with the highest revenues are funded by a combination of state allocation and third-party funds. In other words, the US system includes a large source of revenues that is independent from the number of students and does not depend on political bargaining and which generates the wealth of the top-ranked international universities.

**Fig 6:**
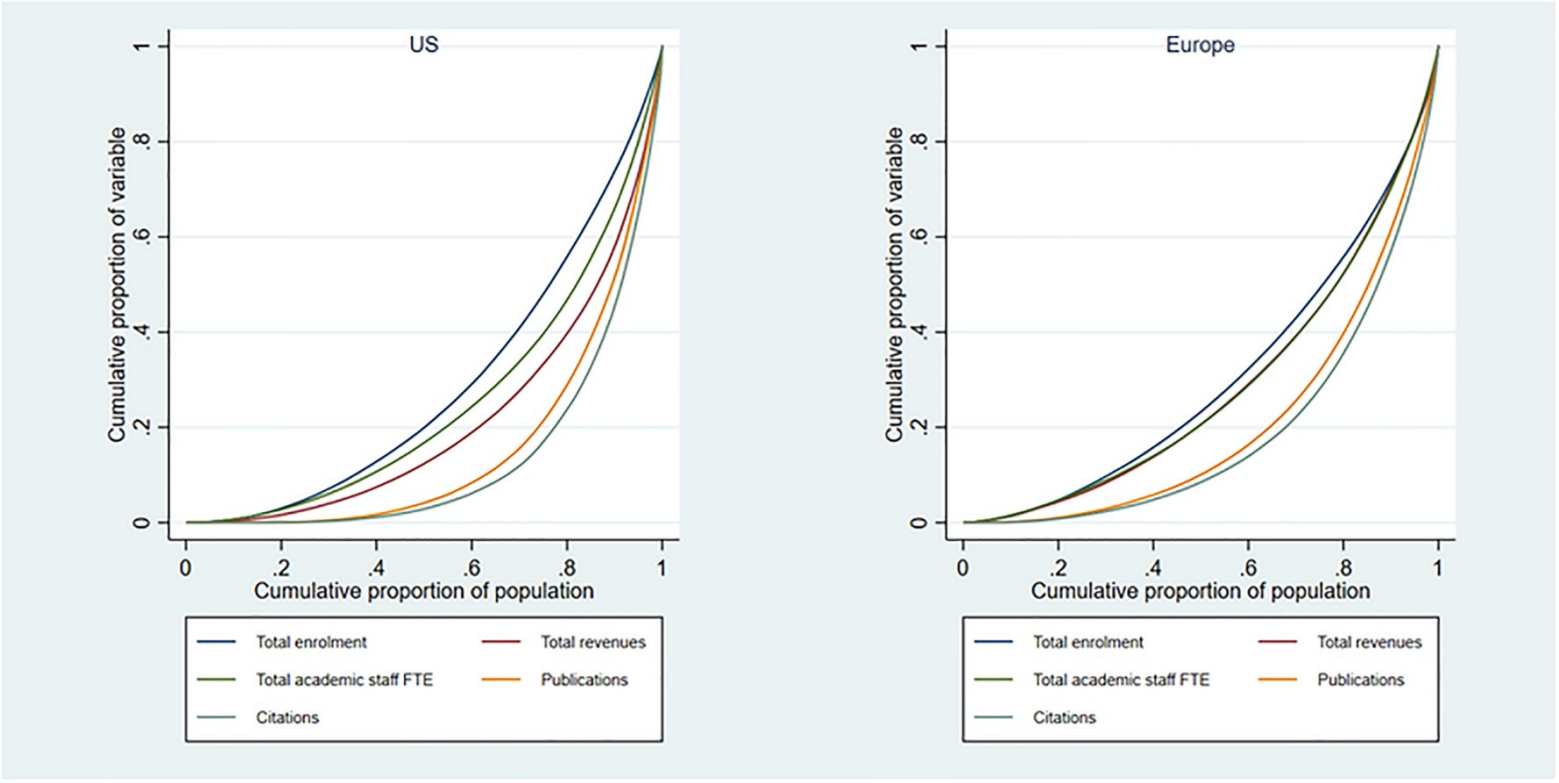
Lorenz curves of the distribution of variables for doctoral universities. N = 930 a) Europe, b) US.

Fourth, there are differences in the respective distribution of input and output within the group of doctoral universities (Fig 6). The level of concentration is similar in both systems for students (the Gini coefficient is 0.419 in the US against 0.383 in Europe) and academic staff (0.492 against 0.427). However, in the US, revenues (0.572 against 0.428), publications (0.691 against 0.584) and citations (0.733 against 0.623) are more concentrated than in Europe when compared to students.

In other words, European HEIs “scale up” with student enrolments, with the distribution of staff and revenues closely following students and with research outputs only moderately more concentrated. On the contrary, revenues are more concentrated than students (and staff) in the US, while publications are far more concentrated. This indicates that the funding mechanisms in the US allows top-ranked universities to receive more resources per unit of staff, without a parallel increase in the number of students. As suggested by our statistical models, this is a powerful driver for achieving international research ‘excellence’.

## Discussion and conclusions

Our results move beyond the current debate on the use of bibliometric indicators for evaluation showing the association of such indicators with the volume of resources [21]. Previous studies of science scaling were focused on the association between publication output and international visibility as measured by citations, but did not include a measure of resourcing [40] [19]. Yet, investigating such a connection is critical for policy evaluation purposes as performance-based allocation of resources [41] represents a core element of the new ‘academic capitalism’ paradigm [42].

Beyond the obvious assumption that more resources produce more output, we have shown that this relationship is tight across a wide range of size and across the two main scientific systems worldwide; further, we have observed super linear scaling both for publications and field-normalized citations, i.e. bibliometric output increases more than proportionally with revenues.

These findings add a further worrisome dimension to the evaluation debate. By and large and especially on the top of the pile, bibliometric indicators and rankings are a richness measure and is questionable whether by orienting their decisions to these indicators policy-makers and stakeholders would do more than enriching the richer, under the presumption of promoting international ‘excellence’. A key component of this process is the existence of a universal (context-free) and measurable definition of ‘excellence’ that might differ from (context-related) quality [13]. Such a measure, like the one conveyed by international rankings, is not necessarily ‘objective’, but nevertheless drives the behavior of the actors, including policy-makers, university managers and scientists themselves [43]. Furthermore, our analysis of financial data shows how these measures are coined to the position of a small set of highly-funded US universities and, therefore, by reproducing the same social norms throughout the higher education system and across countries, contribute to maintain their long-term hegemony [11] [44]. The ‘transatlantic gap’ in research excellence is by and large a ‘resources gap’ generated by the concentration of resources in a few dozens of US universities [12].

We believe that bibliometric indicators do provide valuable information for evaluation purposes at the policy and institutional level. However, we rejoin previous critiques against their de-contextualized usage without taking into account local situations and specificities of scientific fields, countries and institutions [15]. In this respect, our contribution is to demonstrate empirically that the volume of resources represents a key dimension for comparison as so-called scale-free indicators are all size dependent [21]. Our analysis also shows that measures of resources can be used for comparative analysis, albeit with certain limitations, and that the criticism by the bibliometric community that data are not comparable at all is not warranted [45].

Further, we have demonstrated that the strongest association between resources and bibliometric outputs is via additional resources per staff, rather than an increase in the number of faculty. This suggests that a key underlying mechanism explaining the observed patterns is academic mobility, where highly productive scientists move towards the ‘best’ places in terms of ‘excellence’, while in their hiring behavior universities attempt at maximizing ‘excellence’ by investing more resources in few highly productive people [2]. The fact that *at the institutional level* bibliometric indicators provide signals aligned with resources makes this effect pervasive and penalizes highlands of high quality within ‘average’ universities [12].

Our results demonstrate that fighting for the top-positions in international rankings must be associated with the concentration of large amounts of resources in a few places. The analysis of the funding system suggests that this is associated with the long-term construction of institutional structures that allow resources to follow international ‘excellence’ signals [46]. In the US, this was achieved through institutional differentiation and a large amount of resources provided discretionally by private donors, while in Europe, this was achieved only by two countries, i.e. UK with its longstanding tradition of concentrating resources, and Switzerland through the creation of two ‘national’ universities in a federal system. Such processes concern only a tiny minority of institutions and, once established, become self-sustaining thanks to the coupling between ‘excellence’ and resources.

Policy implications are therefore different for the US and for Europe. In the US, promoting international excellence should not be a major focus of public policies as private capital already ensures it; instead, public policies should continue to be focused on widening access and ensuring good quality of education and research throughout the country, following the longstanding tradition of support to colleges and state universities [37]. The increasing privatization of US higher education represents, in this respect, a worrisome tendency [47]. On the contrary, for some (large) European countries currently lacking internationally ‘excellent’ universities, dedicated policies should be designed that trigger the kind of cumulative mechanisms observed in the US, for example by attributing long-term institutional funding. Of course, if this is deemed an important policy objective. To this goal, additional resources would be required as our data show that higher education investment in most European countries is well below the US level. Not only in those countries such as Italy and the UK where in post 2008 crisis budget cuts were implemented, but also in countries such as France and Germany where funding for special excellence initiatives was made available however in a limited scale compared to funding available to top US universities. At the same time, European countries would be well advised to keep their focus on delivery of good quality university education and research at regional level that represents a strength of the European system [48]. Performance-based allocation of funds might contribute to increasing ‘average quality’, but should not be coined towards international excellence as the underlying mechanisms are different. In that respect, there is much to learn in Europe from the US tradition of differentiated policies by types of higher education institutions [49].

In turn, at the institutional level, our results confirm that the battle for international rankings should not be the main concern of most university managers for two reasons: first, this process is driven by largely endogenous mechanisms and, at the least in the short and medium term, there is important inertia that makes it difficult to substantially change the amount and distribution of resources. Second, even in a well-funded system like the US one, this concerns only a handful of universities that account for a tiny proportion of higher education activities, particularly for what concerns education and the contribution to society and economy.

At the same time, in the current evaluative society, higher education institutions cannot avoid comparing themselves. In this respect, our results suggest that the level of resources should be a key criterion for higher education managers to identify peers to compare with, alongside other criteria such as institutional mission and subject areas covered. To that end, institutional rankings based only on size-dependent indicators, should be restructured with a measure of resources or, at least, offer the option of selecting groups of institutions with a similar resource level and provide proper benchmarking strategies oriented by the institutional mission. Our analysis shows that data availability and quality should be no excuse for that.

## Supporting information

S1 Table Mapping scheme for HEI revenues. (DOCX)

S2 Table Descriptive statistics for the doctoral universities sample. (DOCX)

S1 Text Applying the Carnegie classification to our sample. (DOCX)

S2 Text Model diagnostics and robustness tests. (DOCX)
