# Supplementary material for: Scientific output scales with resources. A comparison of US and European universities
Source: PLoS One. 2019 Oct 15;14(10):e0223415. doi: 10.1371/journal.pone.0223415 (PMC6793846; doi:10.1371/journal.pone.0223415)
Supplement: S1 Table — (DOCX) [file pone.0223415.s001.docx]

S1 Table Mapping scheme for HEI revenues

| **ETER Variable** | **IPEDS public HEIs** | **IPEDS private non profit HEIs** | **IPEDS private HEIS** | **ETER** |
| --- | --- | --- | --- | --- |
| Core Budget (public) | Federal Appropriations | |  | Basic government allocation (central or regional) |
|  | State Appropriations | |  |  |
|  | Local Appropriation, Education District taxes, and Similar Support | Local Appropriations |  |  |
|  | Federal Non Operating Grants |  |  |  |
|  | State Non Operating Grants |  |  |  |
|  | Local Non Operating Grants |  |  |  |
| Core budget (private) | Gift (Including Contributions from affiliate Organizations) | Private gifts | Other Revenues | Gifts and donations |
|  | Other Revenues and Additions |  |  |  |
|  | Other Non Operating Revenues |  |  |  |
|  | Sales and Services for Education Activities | | |  |
|  | Other sources - operating |  |  | Interests |
|  | Investment Income | Investment Return | Investment Income and Investment gain (losses) included in net income | Investment income |
| Tuition Fees | Tuition and Fees, after deducting discounts and allowances | Tuition and Fees | Tuition and Fees | Tuition and Fees |
| Third Party | Federal Operating Grants and Contracts | Federal grants and contracts | Federal Appropriations, Grants and Contracts | Public grants and contracts (central, regional ,local) |
|  | State Operating Grants and Contracts | State grants and contracts | State and Local Appropriations, Grants and Contracts |  |
|  | Local Operating Grants and Contracts | Local grants and contracts |  | Grants and contracts from abroad |
|  | Private Operating Grants and Contracts | Private gifts, grants, and contracts | Private Grants and Contracts | Private grants and contracts |
| Unclassifed Revenues | Other sources operating revenues | Other revenues | Other revenues | Other revenues |
| Excluded | Sales and Services of Hospitals | Hospital revenues |  | Healthcare revenues |
|  | Independent Operations Revenue | |  |  |
|  | Sales and Services of Auxiliary Entreprises | Contributions from affiliated entities | Sales and Services of Auxiliary Enterprises | Sales and Services of Auxiliary Enterprises |
