# Supplementary material for: Scientific output scales with resources. A comparison of US and European universities
Source: PLoS One. 2019 Oct 15;14(10):e0223415. doi: 10.1371/journal.pone.0223415 (PMC6793846; doi:10.1371/journal.pone.0223415)
Supplement: S2 Table — (DOCX) [file pone.0223415.s002.docx]

**S2 Table. Descriptive statistics for the doctoral universities sample**

The table below provides descriptive statistics and the correlation table for the main variables used in the regression and for the doctoral universities sample.

Table. Descriptive statistics and Pearson correlation coefficient for the doctoral universities sample

| variable | mean | sd | min | p25 | p50 | p75 | max | Valid N |
| --- | --- | --- | --- | --- | --- | --- | --- | --- |
| ln_budget | 19.09 | 1.16 | 14.04 | 18.45 | 19.17 | 19.84 | 22.32 | 788 |
| ln_students | 9.49 | 0.98 | 3.89 | 9.07 | 9.64 | 10.15 | 11.99 | 929 |
| ln_staff | 6.84 | 0.99 | 3.26 | 6.29 | 6.90 | 7.51 | 8.85 | 814 |
| ln_publications | 6.62 | 1.91 | 1.17 | 5.78 | 6.90 | 8.02 | 10.41 | 893 |
| ln_citations | 6.59 | 2.04 | 1.22 | 5.68 | 6.89 | 8.09 | 10.98 | 891 |
|  |  |  |  |  |  |  |  |  |
|  | US | Europe |  |  |  |  |  |  |
| Region | 366 | 564 |  |  |  |  |  |  |

|  | ln_budget | ln_students | ln_staff | ln_publications | ln_citations |
| --- | --- | --- | --- | --- | --- |
|  |  |  |  |  |  |
| ln_budget | 1 |  |  |  |  |
| ln_students | 0.7066 | 1 |  |  |  |
| ln_staff | 0.8828 | 0.7859 | 1 |  |  |
| ln_publications | 0.8086 | 0.6162 | 0.8485 | 1 |  |
| ln_citations | 0.8142 | 0.6005 | 0.8478 | 0.9943 | 1 |

The distribution of all variables is highly skewed. The Kolmogorov-Smirnov test shows that the normality hypothesis is rejected for all other variables even if logged. Descriptive analysis shows that this is mostly due to a shorter right tail than expected from the lognormal distribution, but most of our data points lie in the central part of the distribution, thereby reducing the risk that the coefficients are strongly influenced by the tails.

As expected, the number of publications and of citations are highly correlated between them and with the budget; budget and staff (logged) are also highly correlated, while the correlation is slightly lower with enrolments.
