# Supplementary material for: Scientific output scales with resources. A comparison of US and European universities
Source: PLoS One. 2019 Oct 15;14(10):e0223415. doi: 10.1371/journal.pone.0223415 (PMC6793846; doi:10.1371/journal.pone.0223415)
Supplement: S1 Text — (DOCX) [file pone.0223415.s003.docx]

**S1 Text. Applying the Carnegie classification to our sample**

The Carnegie classification provides a well-recognized classification of US HEIs (<http://carnegieclassifications.iu.edu/>) that provides a simple division of HEIs mostly based on the type of degrees they award.

For the purposes of this paper, we replicate the classification on our sample of US and European HEIs applying criteria similar to the original Carnegie classification in order to identify the subsample of doctoral universities on which to focus our analysis. Such an approach strongly reduced the heterogeneity of the sample and, specifically, allows excluding specialized research institutions, for which bibliometric indicators are likely to be influenced by the subject domain.

To this aim, we define the following categories:

- Doctoral universities. HEIs with at least 20 ISCED 8 degrees in the year.
- Masters’ colleges and universities. HEIs with less than 20 ISCED8 degrees and at least 50 ISCED 8 degrees.
- Baccalaureate colleges. HEIs with more than 50% of the degrees at level ISCED 6 and with less than 50 ISCED 7 degrees or less than 20 ISCED 8 degrees.
- Baccalaureate/associate colleges. HEIs which can award ISCED 6 degrees, but where more than 50% of the degrees are at level ISCED 5.
- Special focus institutions. HEIs, for which the Herfindal index of the distribution of degrees by educational field (using the OECD-UNESCO fields of education classification) is larger than 0.7, implying that at least 80% of the degrees are in a single educational field.

For the US, these criteria classify 75% of the HEIs in the same category as the 2010 Carnegie classification, with most of the difference being accounted for by a slightly different delimitation of subject fields, by the fact that the Carnegie classification allows for a number of exceptions from the above criteria, and by the fact that our data refer to 2013.

Specifically, for the US, the doctoral university category includes 286 of the 297 doctoral universities in the 2010 original Carnegie classification, in addition to 58 colleges and 16 focused institutions. While the latter can be explained by a different delimitation of subject fields, the former inclusion can be explained by a number of masters’ colleges and universities attaining the threshold for PhD degrees –the 2015 edition of the Carnegie classification counts 329 research universities.

The table below provides descriptive statistics on the resulting classification in the US and in Europe in terms of number of HEIs, staff and enrolments by category.

Table. Carnegie classification applied to the dataset

|  | Europe | | | US | | |
| --- | --- | --- | --- | --- | --- | --- |
|  | N. HEIs | Enrolments | Staff | N. HEIs | Staff | Enrolments |
| Unclassified | 87 | 70,210 | 190 | 231 | 307,332 | 11,991 |
| Doctoral Universities | 564 | 11,200,000 | 671,044 | 366 | 6,291,367 | 469,233 |
| Masters'colleges and universities | 545 | 3,759,457 | 184,660 | 815 | 4,550,288 | 212,263 |
| Baccalaureate colleges | 343 | 789,930 | 32,871 | 637 | 959,374 | 61,304 |
| Baccalaureate/associate colleges | 20 | 58,664 | 2,999 | 487 | 1,049,342 | 33,700 |
| Focused institutions | 684 | 1,179,817 | 70,586 | 751 | 511,493 | 54,239 |
