# Supplementary material for: Scientific output scales with resources. A comparison of US and European universities
Source: PLoS One. 2019 Oct 15;14(10):e0223415. doi: 10.1371/journal.pone.0223415 (PMC6793846; doi:10.1371/journal.pone.0223415)
Supplement: S2 Text — (DOCX) [file pone.0223415.s004.docx]

**S2 Text. Model diagnostics and robustness tests**

We report the diagnostics and robustness tests for the regression model for publications. After the analysis of the residuals we decided to move beyond the standard OLS and consider instead a more robust set of regression methods [27].

Most of the issues that the residual analysis brings us would bias the estimates of the standard errors, but not of the coefficients themselves (with the exception of outliers and leverage points). This is less relevant since our sample covers a large share of the full population of internationally ‘excellent’ universities and, therefore, the estimates we obtain can be considered as very representative of the ‘true’ population values.

**OLS regression diagnostics**

As first step, we perform OLS and run diagnostics on the regression residuals.

Table 1. OLS regression for ln(publications)

| ln_p | Coef. | Std. Err. | p | Coef. | Std. Err. | p |
| --- | --- | --- | --- | --- | --- | --- |
| ln_budgetppp | 1.420 | 0.038 | 0.000 | 1.611 | 0.049 | 0.000 |
| ln_students |  |  |  | -0.141 | 0.061 | 0.021 |
| ssh |  |  |  | -1.201 | 0.198 | 0.000 |
| region |  |  |  | -1.327 | 0.077 | 0.000 |
| _cons | -20.541 | 0.721 | 0.000 | -20.387 | 0.600 | 0.000 |
| N | 763 | | | 759 | | |
| Rsquare | 0.652 | | | 0.785 | | |

**Collinearity**. Variance Inflation Factors range between 2.66, for ln(budget), and 1.1, for the share of students in social sciences and humanities; as shown in Table 1, the coefficient of our main dependent variable is stable against the introduction of the other variables, hence we suggest that collinearity should not be a concern.

**Non-normality**. A shown in Figure 1, residuals are non-normal, mainly because of the tails. In regression analysis, non-normality is however less of a concern since, when the sample size is fairly large, the distribution of the estimates will anyway converge to a normal distribution because of the Central Limit Theorem. In our case, N is much larger than the suggested thresholds in the literature [47], while the residuals’ distribution remains relatively well-behaved.

Figure 1. Regression residuals

For a further robustness test, we perform a regression using a mixtures model, in which it is assumed that the error terms of the regression follows a mixture of normal distributions [48]. This approach has been shown to provide more reliable estimates than OLS when errors are not normally distributed. Results are nearly identical to OLS and, specifically, ln(budget) coefficients remain well above one.

**Influential points and outliers**. We have analyzed influential points and outliers through a leverage vs. squared residuals plot from the OLS regression (see Figure 2). This analysis identifies a number of potential problematic cases. We eventually exclude from the regression eight cases, which are all fairly small universities and, on the aggregate, account for only of 1% of total publications.

Figure 2. Leverage vs. squared residuals plots

**Heteroskedasticity**. The plot of residuals against fitted values (see Figure 3) shows evidence of heteroscedasticity, with the variance being larger for smaller observations. This is expected because smaller universities have less staff and, therefore, more variance in the aggregate number of publications.

Figure 3. Residuals vs. fitted values excluding influential cases

To address this issue, we resort to Feasible Generalized Least Squares (FGLS [28]), a regression model in which the variance is estimated from the data and then used to weight observations in a regression. The model is described in detail in the main text.

While estimates and coefficients remain very similar to OLS, FGLS strongly reduces heteroscedasticity in the residuals (see Figure 4). We therefore use this model for more robust estimates in the main paper. We also tested weighted least squares with 1/ln(budget) as weights, which gave very similar results than FGLS.

Figure 4. Residuals plot, FGLS excluding influential cases

These are weighted residuals, hence the scale is different from Figure 4.

**Linearity.** Linearity is a general concern when analyzing scaling since it is frequent that the scaling coefficient is size-dependent (see [27]). Indeed, an added variable plot shows evidence of non-linearity with the scaling coefficient decreasing with size.

Figure 5. Added variable plot (FGLS excluding influential cases)

To address this issue, we run a quantile regression, which allows investigating the variation of the coefficients by the level of the dependent [29]. As reported in the main text, the scaling coefficient decreases with the dependent, but remains significantly above one even for the largest HEIs.

**Summary of the discussion**. While our data generally do not meet the conditions for an unbiased OLS regression, all tests performed with alternative regression methods provide consistent results, while addressing most of these issues. The two regression models used in the main text, i.e. FGLS and quantile regressions, correct for the two major issues detected, i.e. heteroscedasticity and non-linearity.

In all tests performed, the regression coefficient of ln(budget) remains significantly above one. This, in addition to the fact that our dataset nearly includes the whole population of doctoral universities in the two systems. Therefore, the results should be considered as robust.

**Test with SCIMAGO data**

As reported in Table 2, regression results using SCIMAGO data are nearly identical to the ones reported in the main text using Web of Science data, hence supporting our claim that results are not database-specific.

Table 2. Regresssion results with SCIMAGO data (FGLS, without influential points)

|  | ln(publications SCIMAGO) | | | ln(citations SCIMAGO) | | |
| --- | --- | --- | --- | --- | --- | --- |
|  | Coef. | Std. Err. | p | Coef. | Std. Err. | p |
| ln_budgetppp | 1.382 | 0.039 | 0.000 | 1.699 | 0.048 | 0.000 |
| ln_students | -0.015 | 0.052 | 0.773 | -0.183 | 0.063 | 0.004 |
| ssh | -0.674 | 0.192 | 0.000 | -0.552 | 0.225 | 0.014 |
| region | -1.116 | 0.068 | 0.000 | -1.305 | 0.081 | 0.000 |
| _cons | -15.656 | 0.591 | 0.000 | -19.673 | 0.700 | 0.000 |
| N | 745 | | | 745 | | |
| R-squared | 0.731 | | | 0.723 | | |

**Regional regressions**

As a final test, we perform separate regressions for US and Europe to ascertain whether there are different regional patterns. As shown in Table 3, for the variable of interests, i.e. the volume of the budget, results of the regional regressions are practically identical to the ones in the main regression.

Table 3. Regional regressions (FGLS, without influential points)

| **Europe** | | | | | | |
| --- | --- | --- | --- | --- | --- | --- |
|  | ln(publications) | | | ln(citations) | | |
|  | Coef. | Std. Err. | p | Coef. | Std. Err. | p |
| ln_budgetppp | 1.406 | 0.046 | 0.000 | 1.605 | 0.048 | 0.000 |
| ln_students | -0.130 | 0.055 | 0.019 | -0.231 | 0.060 | 0.000 |
| ssh | -0.022 | 0.186 | 0.907 | 0.236 | 0.204 | 0.249 |
| _cons | -18.398 | 0.662 | 0.000 | -21.308 | 0.725 | 0.000 |
| N | 414 | | | 414 | | |
| R-squared | 0.791 | | | 0.7968 | | |
| **US** | | | | | | |
|  | ln(publications) | | | ln(citations) | | |
|  | Coef. | Std. Err. | p | Coef. | Std. Err. | p |
| ln_budgetppp | 1.440 | 0.054 | 0.000 | 1.646 | 0.055 | 0.000 |
| ln_students | 0.184 | 0.079 | 0.020 | 0.090 | 0.081 | 0.268 |
| ssh | -2.384 | 0.340 | 0.000 | -2.528 | 0.356 | 0.000 |
| _cons | -22.158 | 0.845 | 0.000 | -25.256 | 0.891 | 0.000 |
| N | 337 | | | 336 | | |
| R-squared | 0.837 | | | 0.855 | | |
